# Regulation of the Emissions of the Greenhouse Gas Nitrous Oxide by the Soybean Endosymbiont *Bradyrhizobium diazoefficiens*

**DOI:** 10.3390/ijms23031486

**Published:** 2022-01-27

**Authors:** Emilio Bueno, Daniel Mania, Socorro Mesa, Eulogio J. Bedmar, Åsa Frostegård, Lars R. Bakken, María J. Delgado

**Affiliations:** 1 Estación Experimental del Zaidín, Consejo Superior de Investigaciones Científicas, 18008 Granada, Spain; socorro.mesa@eez.csic.es (S.M.); eulogio.bedmar@eez.csic.es (E.J.B.); 2 Faculty of Chemistry, Biotechnology and Food Sciences, Norwegian University of Life Sciences, P.O. Box 5003, N-1432 Aas, Norway; daniel.mania@nmbu.no (D.M.); asa.frostegard@nmbu.no (Å.F.); lars.bakken@nmbu.no (L.R.B.)

**Keywords:** denitrification, dinitrogen, gene expression, nitric oxide, nitrous oxide reductase

## Abstract

The greenhouse gas nitrous oxide (N_2_O) has strong potential to drive climate change. Soils are a major source of N_2_O, with microbial nitrification and denitrification being the primary processes involved in such emissions. The soybean endosymbiont *Bradyrhizobium diazoefficiens* is a model microorganism to study denitrification, a process that depends on a set of reductases, encoded by the *napEDABC*, *nirK*, *norCBQD*, and *nosRZDYFLX* genes, which sequentially reduce nitrate (NO_3_^−^) to nitrite (NO_2_^−^), nitric oxide (NO), N_2_O, and dinitrogen (N_2_). In this bacterium, the regulatory network and environmental cues governing the expression of denitrification genes rely on the FixK_2_ and NnrR transcriptional regulators. To understand the role of FixK_2_ and NnrR proteins in N_2_O turnover, we monitored real-time kinetics of NO_3_^−^, NO_2_^−^, NO, N_2_O, N_2_, and oxygen (O_2_) in a *fixK*_2_ and *nnrR* mutant using a robotized incubation system. We confirmed that FixK_2_ and NnrR are regulatory determinants essential for NO_3_^−^ respiration and N_2_O reduction. Furthermore, we demonstrated that N_2_O reduction by *B. diazoefficiens* is independent of canonical inducers of denitrification, such as the nitrogen oxide NO_3_^−^, and it is negatively affected by acidic and alkaline conditions. These findings advance the understanding of how specific environmental conditions and two single regulators modulate N_2_O turnover in *B. diazoefficiens*.

## 1. Introduction

Under shortage of oxygen, bacteria can adapt and thrive by two ATP-generating mechanisms: (i) induction of dedicated high-affinity terminal oxidases that permit bacteria to respire oxygen at very low concentrations or (ii) making use of inorganic terminal electron acceptors such as nitrate (NO_3_^−^), which can be reduced through the denitrification pathway to dinitrogen (N_2_) or through dissimilatory nitrate reduction to ammonium (DNRA). Although such anaerobic respiration generates less ATP per mol electron than aerobic respiration, it allows bacteria to grow and persist in the absence of oxygen (O_2_) [1]. Denitrification has been defined as the sequential reduction of NO_3_^−^ or nitrite (NO_2_^−^) to nitric oxide (NO), nitrous oxide (N_2_O), and N_2_ [2]. This process is catalyzed by the periplasmic (Nap) or membrane-bound (Nar) nitrate reductase, nitrite reductases (NirK/NirS), nitric oxide reductases (cNor, qNor, or Cu_A_Nor), and nitrous oxide reductase (N_2_OR) encoded by *nap*/*nar*, *nirK*/*nirS*, *nor*, and *nos* genes, respectively [2,3,4]. In addition to denitrification, multiple pathways for N_2_O generation have been reported, including nitrification, nitrifier denitrification, nitrite oxidation, ammonia oxidation, heterotrophic denitrification, anaerobic ammonium oxidation (anammox), and DNRA [3,5]. N_2_OR is the only known enzyme catalyzing the reduction of N_2_O to N_2_ [6]. Accordingly, expression and activity of N_2_OR are considered natural targets to mitigate N_2_O emissions from agricultural soils [7].

Given the impact of N_2_O as a powerful greenhouse gas in global warming and in depletion of the ozone layer [5,7], understanding its dynamics of production/reduction in soils and aquatic environments has become a priority. In fact, the application of synthetic nitrogen (N) fertilizers to agricultural soils, as well as local oxygen concentrations, water content, carbon availability, and pH, greatly affect N_2_O emissions from soils and aquatic ecosystems [7,8,9].

Many legumes stablish symbiotic associations with soil bacteria, collectively termed “rhizobia”, which fix nitrogen in so-called root nodules on legume roots and on the stems of some aquatic legumes. Following invasion of the plant cells via a complex signaling pathway between bacteria and plant, rhizobia stop dividing and undergo differentiation into nitrogen-fixing bacteroids, at which point the nitrogenase complex reduces atmospheric N_2_ into biologically useful forms in a process called “Biological N_2_ Fixation”. Consequently, cultivation of legumes can reduce the need for environmentally polluting synthetic nitrogen fertilizers, thus decreasing N_2_O emissions as well as protecting ground water from toxicity while improving soil fertility. However, legume crops can also contribute to N_2_O emissions in several ways: (i) by biologically fixed N_2_ being converted to NO and N_2_O through nitrification and denitrification [10]; (ii) by providing N-rich residues for decomposition [11], and (iii) directly by some rhizobia that can denitrify under free-living conditions or in symbiotic association with legumes [12,13]. In this context, one strategy to reduce N_2_O emissions from legume crops is to use as inoculants rhizobia strains with high N_2_OR activity. In fact, it has been shown that N_2_O emissions from soybean crops can be reduced by inoculating legumes with strains of the soybean endosymbiont *Bradyrhizobium diazoefficiens* that overexpress N_2_OR [14,15].

**Figure 1:**
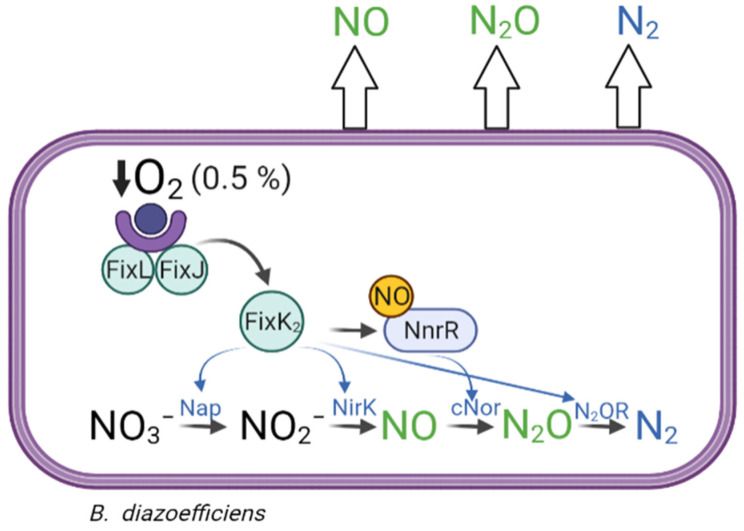
Schematic representation of the denitrification process and its regulation in *Bradyrhizobium diazoefficiens*. *B. diazoefficiens* can reduce nitrate (NO_3_^−^) to nitrite (NO_2_^−^), nitric oxide (NO), nitrous oxide (N_2_O), and dinitrogen (N_2_) by the periplasmic nitrate reductase (Nap), copper-containing nitrite reductase (NirK), nitric oxide reductase type *c* (cNor), and nitrous oxide reductase (N_2_OR) enzymes, respectively. In *B. diazoefficiens,* expression of denitrification enzymes is tightly regulated by the FixLJ, FixK_2_, and NnrR regulatory proteins (see Introduction for further details). However, despite the coordinated activation of each reductase, environmental unfriendly gases such as NO and N_2_O can leak from denitrification and be released to the atmosphere.

*B. diazoefficiens* is considered a model bacterium to study denitrification in rhizobia, since it is the only rhizobial species that, in addition to fixing N_2_, has the ability to grow under anoxic conditions by reducing NO_3_^−^ through the complete denitrification pathway, a process widely studied in this bacterium both in free-living conditions and in symbiosis with soybeans [12,13,16]. *B.* *diazoefficiens* possesses the complete set of *napEDABC*, *nirK*, *norCBQD*, and *nosRZDFYLX* denitrification genes [12], which encode the periplasmic nitrate reductase (Nap), copper-containing nitrite reductase (NirK), nitric oxide reductase type *c* (cNor), and nitrous oxide reductase (N_2_OR), respectively (Figure 1). Like many other denitrifiers, expression of denitrification genes in *B. diazoefficiens* requires both oxygen limitation and the presence of NO_3_^−^ or a nitrogen oxide (NOx) derived from its reduction. The response to low oxygen (≤0.5% O_2_ in the gas phase, i.e., ≤5–10 µM O_2_) is mediated by the FixLJ-FixK_2_-NnrR regulatory cascade [3], in which the response regulator FixJ in its active phosphorylated form induces the expression of several genes, including *fixK*_2_, which encodes the transcriptional regulator FixK_2_ (Figure 1). This protein induces the expression of more than 300 genes, including genes associated with microoxic metabolism (*fixNOQP*), denitrification genes (*napEDABC, nirK, norCBQD*, and *nosRZDFYLX*), and regulatory genes (*rpoN_1_*, *fixK_1_*_,_ and *nnrR*) [12,17,18]. It has also been demonstrated that expression of *napEDABC, nirK*, and *nosRZDFYLX* genes requires microoxic conditions and directly depends on FixK_2_, while expression of *norCBQD* genes relies on NO, being the transcriptional regulator NnrR the candidate that directly interacts with *norCBQD* promoter [19,20] (Figure 1).

Although much is known about the role of FixK_2_ and NnrR in the regulation of denitrification in *B. diazoefficiens,* this knowledge needs to be extended to include relevant physiological conditions that this bacterium is expected to meet in nature. In the present work, we investigated the dynamics of N_2_O balance by FixK_2_ and NnrR as well as the influence of specific environmental conditions such as the presence of nitrogen oxides, O_2_ concentration, pH, and the redox state of C-sources.

## 2. Results

### 2.1. N_2_O Emissions by B. diazoefficiens 110spc4 Depend on the FixK_2_ and NnrR Regulatory Proteins

**Figure 2:**
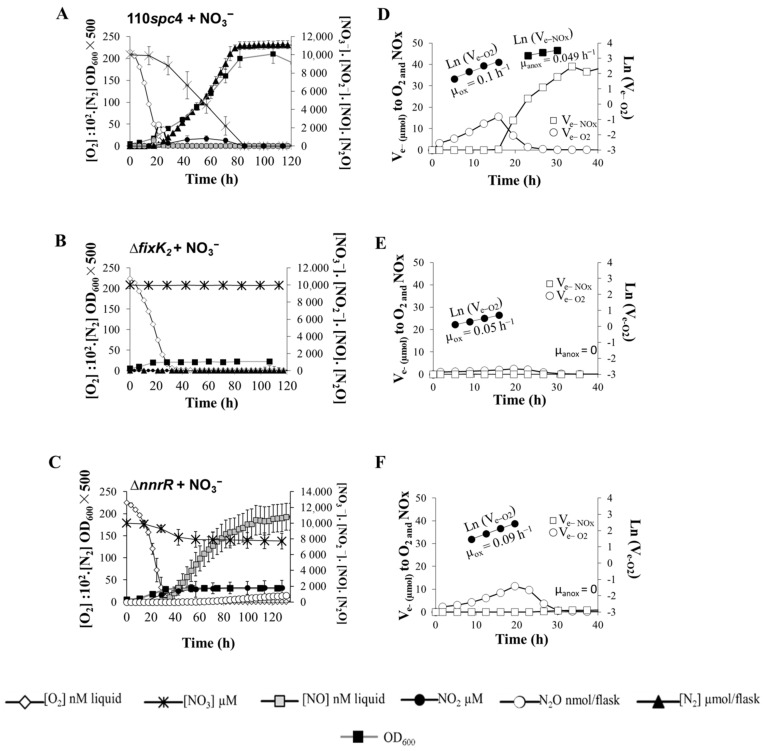
Denitrification phenotypes of the parental strain *B. diazoefficiens* 110*spc*4 (**A**) and the two mutant strains ∆*fixK*_2_ (**B**) and ∆*nnrR* (**C**). (**A**–**C**) measurement of O_2_ and NO_3_^−^ respiration, concentrations of denitrifying intermediaries (NO_2_^−^, NO, N_2_O, N_2_), and bacterial growth (OD_600_) yielded from such dynamics. (**D**–**F**) electron flow rates to O_2_ and nitrogen oxides (NO_x_). Cells were incubated with 2% O_2_ and 10 mM NO_3_^−^ as oxic and anoxic respiratory substrates, respectively. O_2_, NOx concentrations, and bacterial growth were monitored by automatic sampling from headspace and liquid phase. See Figure S1 to visualize individual gases’ dynamics from (**A**,**C**). Data are the means and standard deviations of at least three different cultures.

*B. diazoefficiens* strains were raised oxically under vigorous stirring, and aliquots were inoculated into culture vials to an initial OD_600_ of 0.01 (8 × 10^6^ cells mL^−1^). Next, 2% O_2_ and 10 mM NO_3_^−^ were added as oxic and anoxic electron acceptors, respectively. Figure 2 and Figure S1 show the O_2_, NO_3_^−^, NO_2_^−^, NO, N_2_O, and N_2_ concentrations throughout the 120 h incubation of *B. diazoefficiens* 110*spc*4 (wild type) (Figure 2A and Figure S1A) and *fixK*_2_ (Figure 2B) and *nnrR* (Figure 2C and Figure S1B) mutant strains. *B. diazoefficiens* wild type consumed O_2_ within 28 h, and bacterial OD_600_ increased following O_2_ depletion.

Rates of O_2_ consumption for each time increment between two samplings were taken to calculate electron (e^−^) flow rates to oxygen (V_e−O2_). As shown in Figure 2D, V_e−O2_ increased exponentially in the wild type during the first 16 h and declined gradually in response to diminishing O_2_ concentrations. The increase in electron flow can be taken as an indirect measure of growth (µ_ox_) [21]. Thus, the apparent µ_ox_ estimated by linear regression of ln (V_e_−_O2_) against time was 0.10 (±0.03) h^−1^ (Figure 2D, Table 1A). Final OD_600_ resulting from the consumption of 2% O_2_ was 0.080 (±0.005) (6.40 × 10^7^ cells mL^−1^, Table 1B), equivalent to a yield of 13.3 (±1.1) cells pmol^−1^ e^−^ to O_2_ (Table 1A). Remarkably, in contrast to the fast depletion of O_2_ observed in the parental strain, the capacity to consume O_2_ in the *fixK*_2_ and *nnrR* mutant strains was slightly reduced (Figure 2A–C). In the case of the *fixK*_2_ mutant, V_e_−_O2_ increased exponentially throughout the first 19 h and then declined gradually (Figure 2E). As shown in Table 1A, the apparent µ_ox_ was 0.055 (± 0.008) (Figure 2E, Table 1A). The final OD_600_ from O_2_ respiration was 0.044 (±0.003) (Table 1B), resulting in a yield of 6.6 (±0.3) cells pmol^−1^ e^−^ to O_2_ (Table 1A). In the *nnrR* mutant, electron flow to O_2_ increased exponentially throughout the first 19 h with an apparent µ_ox_ of 0.090 (±0.004) and then decreased slowly (Figure 2F). The final OD_600_ during oxic phase was 0.079 (±0.001) (Table 1B), with a subsequent yield of 13.1 (±0.6) cells pmol^−1^ e^−^ to O_2_ (Table 1A).

Initiation of denitrification in the parental strain, hallmarked by the reduction of NO_3_^−^ and transient emissions of NO and N_2_O (Figure 2A and Figure S1A), was observed at O_2_ concentrations of ≤5 (±0.3) μM O_2_ (Figure 2A and Figure S1A and Table 1B) after 17 h of incubation. Rapidly, N_2_ production was detected as individual final product from NO_3_^−^ denitrification, with 100% of NO_3_^−^ being converted to N_2_ within 80 h of growth. NO_2_^−^ accumulated for a longer period than NO and N_2_O; however, its concentration was maintained at low levels until it was totally reduced to its depletion (Figure S1A).

As shown in Figure 2A, growth of *B. diazoefficiens* increased proportionally with NO_3_^−^ respiration. Interestingly, the parental strain was able to derive electrons to NO_3_^−^ reduction during the oxic phase before O_2_ was depleted, thus securing the transition from aerobic to anaerobic respiration and avoiding anaerobic entrapment (Figure 2D). Electron flow to NO_3_^−^ increased exponentially during the anoxic phase, with an estimated growth rate (µ_anox_) of 0.049 (±0.004) h^−1^ (Figure 2D; Table 1A). The final OD_600_ was 0.40 (±0.05) (Table 1B) and cell yield resulting from NO_3_^−^ respiration (5.1 (±0.8) cells pmol^−1^ e^−^ to NO_3_^−^) (Table 1A) was around 2.6-fold lower than that observed during oxic respiration.

In contrast to the competent transition from aerobic to anaerobic NO_3_^−^ respiration by the parental strain, the *fixK*_2_ mutant strain was unable to shift to anaerobic respiration (Figure 2B), and following the oxygen depletion, the electron flow dropped drastically to zero (Figure 2E). Remarkably, ∆*nnrR* was able to initiate denitrification at O_2_ concentrations of ≤3.3 μM (±2.3) after 31 h incubation but was unable to consume NO derived from NO_2_^−^ reduction, and consequently, NO accumulated in the headspace of the incubation medium (Figure 2C and Figure S1B and Table 1B). This accumulation of NO probably inhibited NO_3_^−^ reduction and concomitant growth.

### 2.2. N_2_O Reduction by B. diazoefficiens 110spc4 Relies on the FixK_2_ and NnrR Regulatory Proteins in a Nitrogen-Oxides-Independent Manner

**Figure 3:**
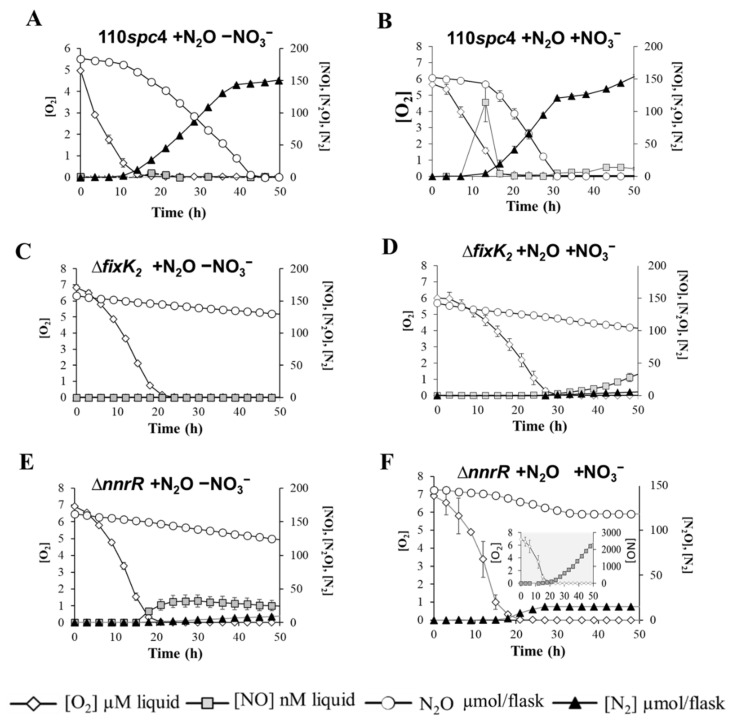
Impact of FixK_2_ and NnrR inactivation on N_2_O consumption. Measurement of O_2_ and N_2_O respiration and concentrations of NO and N_2_. *B. diazoefficiens* 110*spc*4 parental strain (**A**,**B**) and *fixK*_2_ (**C**,**D**) and *nnrR* (**E**,**F**) mutant strains were incubated in vials containing 0.5% O_2_ and 5% N_2_O as oxic respiratory and anoxic respiratory substrates, respectively. In addition, a second set of vials were also supplemented with 10 mM NO_3_^−^ (**B**,**D**,**F**) as anoxic respiratory substrate. The gradual decline in N_2_O concentration in (**C**,**D**,**E**) corresponds to dilution of headspace gases due to sampling. Data are the means and standard deviations of at least three different cultures.

Transient detection of N_2_O in *B. diazoefficiens* wild type and inhibition of the denitrification process in ∆*fixK*_2_ and ∆*nnrR* strains precluded comparison of their N_2_O reduction capacities. Thus, to specifically assess the capacity of *B. diazoefficiens* wild type and *fixK*_2_ and *nnrR* mutant strains to consume N_2_O, we undertook a complementary approach. We supplied *B. diazoefficiens* bacterial cells with artificial N_2_O and analyzed their capacity to consume it. N_2_O reduction and subsequent N_2_ production were monitored in vials containing 5% N_2_O injected into the headspace. In addition, to study the impact that the presence of nitrogen oxides (NO_x_) might exert on N_2_O reduction, we also examined *B. diazoefficiens*’ capacity to consume N_2_O in the absence (Figure 3A,C,E) and in the presence (Figure 3B,D,F) of NO_3_^−^. In addition to N_2_O, 0.5% O_2_ was also added to the headspace as aerobic respiratory substrate due to the incapacity of *B. diazoefficiens* to initiate growth in the total absence of O_2_ (data not shown). Regardless of the presence of NO_3_^−^, externally supplied N_2_O was rapidly reduced to N_2_ by the parental strain until its complete depletion (Figure 3A,B). The final OD_600_ (Table 2B) and yield (Table 2A) of *B. diazoefficiens* parental cells were also monitored upon N_2_O consumption, and we found that both growth parameters were significantly enhanced when the bacterium was simultaneously incubated with both alternative electron acceptors, N_2_O and NO_3_^−^ (Table 2A,B).

In the absence of NO_3_^−^, N_2_O reduction was initiated at O_2_ concentrations of ≤0.66 (±0.05) µM in the parental strain (Figure 3A; Table 2B). Under these conditions, electron flow to N_2_O increased with an apparent growth rate (µ_N2O_) of 0.028 (±0.002) h^−1^ estimated by linear regression of ln (V_e−N2O_) against time (Figure S2A, Table 2A). Electron flow rates to N_2_O remained unnoticeable during the first 5 h of oxic respiration; however, they increased exponentially after 8 h when electron flow to O_2_ was high. Similar to that which was previously observed during anaerobic NO_3_^−^ respiration, this premature induction of the N_2_OR in the presence of O_2_ might be a mechanism to elude anoxia entrapment during the transition from oxic to anoxic conditions.

When NO_3_^−^ was present, initiation of denitrification, hallmarked by a transient emission of NO (113 nM ± 30), took place after 7 h incubation under nearly anaerobic conditions (O_2_ concentrations of 1.5 (±0.15) μM) (Table 2B and Figure 3B, respectively), and it preceded induction of N_2_O consumption. In fact, N_2_O reduction was initiated at lower O_2_ concentrations of ≤0.15 (±0.05) µM (Figure 3B; Table 2B), indicating the *B. diazoefficiens*’ preference for NO_3_^−^ as terminal electron acceptor. Estimated anaerobic growth rate supported by N_2_O in the presence of NO_3_^−^ was µ_N2O_ = 0.046 (±0.003) h^−1^ (Figure S2B, Table 2A). Equivalently to that which was observed in the absence of NO_3_^−^, electron flow to N_2_O reduction occurred during active O_2_ respiration after 5 h incubation in the presence of NO_3_^−^ (Figure S2B).

Strikingly, *B. diazoefficiens* strains lacking the regulatory transcriptional factors FixK_2_ or NnrR were severely impaired in N_2_O consumption capacity and growth (Figure 3C–F, Table 2A,B). Despite ∆*fixK*_2_ being unable to reduce N_2_O and grow either in the absence or the presence of NO_3_^−^, traces of NO gas were detected after 40 h incubation with NO_3_^−^ (Figure 3D), but such residual respiratory activity was not coupled to growth. A mutant strain defective in the *nnrR* gene was significantly defective in its capacity to reduce N_2_O when incubated without NO_3_^−^ (only 8 (±0.5)% of N_2_O was reduced to N_2_), likely due to its incapacity to detoxify NO, which permanently accumulated in the medium up to 32.2 (±8.8) nM (Figure 3E, Table 2B). The presence of NO_3_^−^ slightly induced N_2_O reduction by the *nnrR* mutant (12.5 (±2.1)% of N_2_O was reduced to N_2_) at O_2_ concentrations of 0.8 (±0.3) μM after 13 h incubation (Figure 3F, Table 2B). However, under these conditions, NO_3_^−^ in the medium was further reduced to NO, which was accumulated after 20 h incubation reaching levels up to ~2 µM after 50 h incubation (Figure 3F, insert, Table 2B).

Our results explain that *B. diazoefficiens* can co-respire NO_3_^−^ and N_2_O and that activation of the N_2_O reductase relies on the FixK_2_ and NnrR regulatory proteins, independently of the presence of nitrogen oxides. Lastly, we also found that N_2_O reductase activity in *B. diazoefficiens* is highly sensitive to accumulation of endogenous NO derived from NO_3_^−^ respiration, further supporting the importance of coordinated activation of denitrifying reductases by the FixK_2_ and NnrR regulators.

### 2.3. Acidic and Alkaline pHs Impair N_2_O Reduction by B. diazoefficiens 110spc4

**Figure 4:**
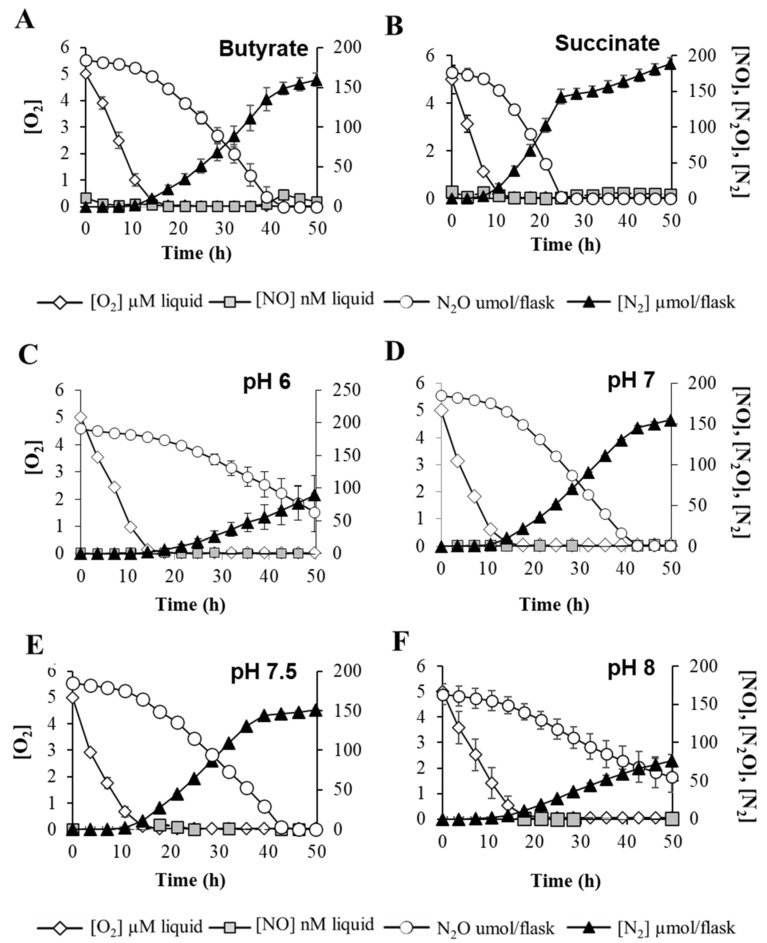
Impact of C-source and pH on N_2_O consumption. Measurement of O_2_ and N_2_O respiration and concentrations of NO and N_2_. *B. diazoefficiens* 110*spc*4 parental strain was incubated in vials containing 0.5% O_2_, 5% N_2_O, and 10 mM NO_3_^−^ as substrates for aerobic and anaerobic respiration, respectively. C-sources (**A**,**B**) and pH (**C**–**F**) of the growth medium were modified as shown on the graphs (see Material and Methods for further details). O_2_ and NOx concentrations were monitored by automatic sampling from headspace phase. Data are the means and standard deviations of at least three different cultures.

To further elucidate how environmental cues prevailing in *B. diazoefficiens* niches might modulate N_2_O reduction, we monitored the expression of *nosRZDFYLX* genes and the capacity of *B. diazoefficiens* to reduce N_2_O in the presence of C-substrates commonly encountered in a plant’s rhizosphere [22], such as succinate, which generates 2 mol e^−^ per C-mol oxidized, and butyrate, which generates 5 mol e^−^ per C-mol oxidized. Interestingly, such C-sources did not affect expression of the *nos* operon (Figure S3A). Next, we analyzed N_2_O consumption by *B. diazoefficiens* determined as changes in N_2_O concentration in the headspace of vials containing 0.5% O_2_ plus 5% N_2_O inoculated with aerobically raised bacterial cells. Monitoring O_2_ uptake by *B. diazoefficiens* during the oxic phase also allowed us to evaluate any effect of C-source on bacterial metabolism/energetic that could subsequently alter N_2_O respiration. Despite N_2_O consumption was delayed around 20 h in the presence of butyrate compared to succinate (Figure 4A,B), such impairment could be attributed to a general metabolic defect, as oxygen consumption during the first hours of growth also was attenuated in that C-source. Further metabolic analyses are required to shed light on this respiratory inhibition induced by reduced C-sources.

To understand if local changes in soil pH might affect N_2_O emissions from *B. diazoefficiens,* we also examined N_2_OR gene expression and N_2_O consumption in cells incubated at different pHs. As shown in Figure S3B, *nos* expression levels after 20 and 30 h incubation were not affected by different pH levels. Interestingly, while O_2_ consumption was similar at different pH levels, N_2_O reduction was strongly diminished at pH 6 and 8 (Figure 4C–F). These findings imply that, in addition to the impact of FixK_2_ and NnrR regulatory proteins on N_2_O reduction, relevant environmental factors such as pH importantly influence dynamics of N_2_O reduction by *B. diazoefficiens*.

## 3. Discussion

Given the damaging effect of N_2_O on climate, strategies to mitigate N_2_O emissions arising from intensive agricultural practices must be developed. These strategies include: (i) management of soil chemistry and microbiology to ensure that bacterial denitrification proceeds to completion, forming N_2_; (ii) promotion of sustainable agriculture, i.e., obtaining higher output from the same cultivated area of land; (iii) a better understanding of the environmental and regulatory factors that contribute to the generation and consumption of biological N_2_O; and (iv) reducing the dependence on fertilizers by using engineered crops that fix dinitrogen themselves or, alternatively, through application of nitrogen-fixing bacteria to legume crops. Despite the latter being one of the most promising alternatives to reduce N_2_O emissions, denitrification within endosymbiotic and free-living rhizobia released from nodules also contributes to the emission of N_2_O [10,13,16,23,24]; therefore, a better knowledge of the environmental and cellular factors controlling rhizobial denitrification is required.

Environmental cues (oxygen tensions and nitrogen oxides) and regulatory proteins (FixK_2_ and NnrR) governing denitrification in *B. diazoefficiens* are well-known [19,20,25] (Figure 1). In this work, we have validated, under physiological conditions, the importance of the FixK_2_ and NnrR transcription factors in real-time N_2_O dynamics using a robotized incubation system. Hence, we were able to simultaneously monitor changes in O_2_, NO_3_^−^, NO_2_^−^ NO, N_2_O, and N_2_ concentration during the transition from aerobic to anaerobic respiration in *B. diazoefficiens* wild type and *fixK*_2_ and *nnrR* regulatory mutants. In addition, we also performed precise estimations of growth parameters (i.e., μ, yield) and defined accurately the O_2_ concentrations in which each step of the denitrification process is triggered. Therefore, we were able to determine that the denitrification process in *B. diazoefficiens* occurs at O_2_ concentrations of ≤5 (±0.3) μM. This concomitant induction of the denitrifying machinery with oxic respiration ensures a smooth and efficient transition from aerobic to anaerobic respiration, avoiding depression of electron flow when O_2_ is scarce (Figure 2A,D). A similar scenario was previously observed in the plant pathogen *Agrobacterium tumefaciens* [26]. In contrast to the early induction of the denitrification process found in these plant-interacting bacteria, *Paracoccus denitrificans* initiates transcription of nitrite reductase very late, resulting in entrapment of the majority of cells in anoxia [27].

We have also demonstrated, for the first time, that *B. diazoefficiens* 110*spc*4 is an efficient denitrifier, as it is able to transform 100% of NO_3_^−^ to N_2_ (Figure 2A and Figure S1A). Interestingly, emission of N_2_O was detected at an early peak in O_2_ concentration of ≤5 μM (Figure 2A and Figure S1A and Table 2) during the transition from aerobic to anaerobic respiration, but the bacteria rapidly reduced its concentration, keeping it under very low levels (~2 ppm in headspace; 40 nM in the liquid). Collectively, these results reveal that denitrification in *B. diazoefficiens* 110*spc*4 emits marginal amounts of N_2_O, implying, as demonstrated by Mania et al., (2020) [28] and by Gao et al., (2021) [29], that bradyrhizobia can constitute a strong sink of the N_2_O released by neighboring organisms in the soil. Such denitrifying activity depends on coordinated activities of FixK_2_ and NnrR regulatory proteins. The tight control on emission of N_2_O and other denitrifying gases has been previously described in diverse bacterial species [26,27,28,29].

Although NO is a key signal molecule for the regulation of many processes, at high concentrations it exerts toxicity at different cellular levels [30,31,32]. Consequently, bacteria employ dedicated regulatory systems to keep NO at very low concentrations. Strikingly, we found that NO levels in *B. diazoefficiens* cultures reached very high concentrations (~600 nM) (Table 1B). Similarly, *A. tumefaciens* also accumulates large amounts of NO; however, those NO concentrations were not detrimental for this closely related rhizobium [26]. Conversely, *P. denitrificans, Pseudomonas aerofaciens*, and strains from the genus *Thauera* present a relatively tight control of NO production, maintaining NO concentration lower than 10–50 nM [21,27,33]. Although the reason for these differences in control of and tolerance to NO concentrations is unknown, it might arise from differential selective pressures exhibited by their ecological niches. Hence, while *P. denitrificans*, *P. aerofaciens*, and *Thauera* genus comprise bacteria that exist under free-living conditions, *A. tumefaciens* and *B. diazoefficiens* are bacteria that can interact with plants establishing pathogenic and symbiotic relationship, respectively. During its interaction with plants, *A. tumefaciens* might face diverse host defense systems such as NO production. Thus, a high tolerance to NO might confer a certain fitness advantage in respect to other soil competitors. NO is also known to be produced by plants in early stages during its interaction with nitrogen-fixing bacteria, as well as within the mature nodule [34,35,36,37]. In this context, symbiotic bacteria might require higher tolerance to NO to establish a productive symbiotic interaction with the plant.

In contrast to the efficient denitrifying capacity of *B. diazoefficens* wild type, we found that the *fixK*_2_ mutant was unable to initiate NO_3_^−^ reduction. On the contrary, *nnrR* mutant cells were able to initiate the reduction of NO_3_^−^ to NO_2_^−^ and to NO; however, they were entrapped into anoxia due to accumulation of toxic concentrations of NO (Figure 2B,C). This disparate response of *fixK*_2_ and *nnrR* mutants confirms previous results in vitro, where we demonstrated that FixK_2_ directly controls the expression of *napEDABC*, *nirK*, and *nosRZDFYLX* genes in response to microoxic conditions and NnrR is the regulator that directly interacts with *norCBQD* promoter in response to NO [19,20]. Similar denitrification phenotypes were observed in *P. denitrificans* mutants deficient in the O_2_ and NO sensors FnrP and NNR, respectively [38].

Since NO_3_^−^ reduction in ∆*fixK*_2_ and ∆*nnrR* was abrogated, we could not valuate their capacity to produce or consume N_2_O resulting from NO_3_^−^ reduction. To achieve this goal, we incubated the cells in the presence of N_2_O, and we analyzed N_2_O and N_2_ fluxes. In the parental strain *B. diazoefficiens* 110*spc*4, N_2_O reduction was initiated at O_2_ concentrations of 0.15 (±0.05) and 0.66 (±0.05) μM in the presence and in the absence of NO_3_^−^, respectively. In contrast to the low O_2_ concentration required to trigger N_2_O consumption in *B. diazoefficiens*, in other rhizobia species such as *Ensifer meliloti* strain 1021, N_2_O consumption was initiated at O_2_ concentrations of 8 μM [39], indicating that *B. diazoefficiens* presents a N_2_OR more sensitive to O_2_ than other closely related rhizobial species.

In addition to microoxia, the nitrogen oxide NO_3_^−^ and its reduction products NO_2_^−^ or NO are considered essential inducers of denitrification in *B. diazoefficiens* [3,20]. Remarkably, we demonstrated in this work that N_2_O reduction in this bacterium was triggered in the absence of NO_3_^−^. Supporting our observations, it has been previously reported that microoxia is the main signal of expression of *B. diazoefficiens nosRZDYFLX* genes and N_2_OR activity [19]. This independence from NO_3_^−^ was also reported in *E. meliloti* [39] and *P. denitrificans* [33].

When N_2_O was externally supplied, the parental strain reduced 100% of N_2_O to N_2_. In contrast, the N_2_O-reducing capacity of the *fixK*_2_ mutant was totally abolished in a medium without or with NO_3_^−^. However, *nnrR* mutant cells were able to reduce some N_2_O to N_2_ in the absence or in the presence of NO_3_^−^ (8 (±0.5)% and 12.5 (±2.1)%, respectively) (Table 2B). These results confirm previous reports that propose FixK_2_ but not NnrR as the main transcriptional activator of the *nosRZDYFLX* genes [19]. In contrast to the disparate contribution of FixK_2_ and NnrR observed in our studies, it has been proposed that the homologous regulators of *P. denitrificans* FnrP and NNR contribute equally to N_2_OR induction [38,40,41]. Interestingly, cultures from ∆*nnrR*, with or without nitrate, showed a weak N_2_OR activity. In contrast to the transient accumulation of NO detected in cultures from the WT strain with NO_3_^−^ (Figure 3B), the ∆*nnrR* mutant seems to be unable to detoxify NO, which remains permanently in the medium throughout the incubation (Figure 3F, insert). This long-lasting accumulation of NO was also observed when the medium was not supplemented with nitrate (Figure 3E). This NO may arise from traces of nitrate present in this medium (~50–100 µM, data not shown). The permanent accumulation of NO (32 nM) in ∆*nnrR* cells incubated without nitrate or when they were incubated with nitrate (~2 µM) might impair N_2_O reduction of the *B. diazoefficiens* ∆*nnrR* mutant.

An optimal management of soils is crucial to induce N_2_OR activity. In this context, it has been reported that maintaining soil pH at high ranges promotes N_2_OR activity. This strategy is based on the reported sensitivity of the N_2_O reductase activity to low pH in denitrifying bacteria [33,39], in bacterial communities extracted from soils and in intact soils [42]. Carbon availability also has an important role in N_2_O emissions from soils [43]. However, how specific forms of reductants might affect expression and activity of N_2_OR is largely unexplored. To study ecologically relevant environmental factors that could influence *B. diazoefficiens* N_2_OR expression and activity, we analyzed the expression of a *nosR-lacZ* transcriptional fusion as well as N_2_OR activity by monitoring N_2_O consumption, in the presence of reduced or oxidized C-sources such as butyrate or succinate and at different pH values. Despite the fact that expression of the *nos* genes was not affected by any of the conditions tested, N_2_OR activity was significantly attenuated when *B. diazoefficiens* cells were incubated under acidic and alkaline pHs (i.e., pH 6 and pH 8). Moreover, N_2_OR activity was also negatively affected when cells were incubated with reduced C-sources. However, reduced C-sources also affected oxygen consumption, which may indicate a general defect in bacterial metabolism when using such a C-source.

Confirming these observations, low pH had little effect on the transcription of the *nosZ* gene in *P. denitrificans* [33]. Instead, the enzymatic rate of N_2_O reduction was significantly attenuated at low pH levels, suggesting that environmental pH may have a direct posttranslational effect on the assembly and/or activity of the N_2_OR holoenzyme. Consistent with these findings, pH did not affect gene expression of *Marinobacter hydrocarbonoclasticus* N_2_OR genes; however, the amount of N_2_O reductase isolated from cells grown at pH 6.5 was lower than that at pH 7.5 and 8.5, pointing to a post-transcriptional regulation [44]. Indeed, biochemical studies of the *M. hydrocarbonoclasticus* N_2_OR revealed that redox properties of its catalytic site are significantly altered by changes in pH values ranging from 6.5 to 8.5 [44]. Similarly, as observed in *B. diazoefficiens*, an inhibitory effect of reduced carbon sources such as butyrate or low pH on N_2_OR activity was already observed in *E. meliloti* [39]. In contrast to *E. meliloti* [39] and *M. hydrocarbonoclasticus* [44], in our work, *B. diazoefficiens* N_2_OR was also inhibited at a high pH, buttressing the importance of controlling’ soils pH regarding N_2_O emissions. Such sensitivity of *B. diazoefficiens* N_2_OR to high pH is currently under investigation.

Altogether, these observations expand the knowledge of the regulatory and environmental factors that control N_2_O emissions by bacterial species associated with legumes. This information should be taken into consideration when developing new programs to manage N_2_O emissions from legume crops.

## 4. Materials and Methods

### 4.1. Bacterial Strains and Growth Conditions

*Bradyrhizobium diazoefficiens* 110*spc*4 [45] and Δ*fixK*_2_::Ω and Δ*nnrR*:: *aphII* mutant strains [20] were used in this work. To analyze expression of the *nosRZDYFLX* genes, a *B. diazoefficiens* strain (110spc4-BG0306) containing a chromosomally integrated transcriptional fusion within the *nosRZDYFLX* genes promoter and the *lacZ* reporter gene was used [19]. *B. diazoefficiens* strains were firstly grown aerobically in 120 mL serum vials each containing a magnetic stirring bar and 50 mL of Peptone-Salts-Yeast extract (PSY) complete medium [46] at 30 °C. To analyze anaerobic growth from *B. diazoefficiens*, aliquots from aerobic cultures raised under vigorous stirring to avoid anoxic microzones by cells aggregation were transferred to vials with minimal defined Bergersen’s medium [47]. Oxygen from vials was removed by 6 cycles of air evacuation for 360 s and helium (He) filling for 40 s. Influence of pH on N_2_O consumption was analyzed by cultivating *B. diazoefficiens* under N_2_O respiring conditions in minimally defined medium buffered with 50 mM phosphate buffer at pH 6, 7, 7.5, and 8. In all the treatments, the headspace was filled with an initial concentration of O_2_ of 0.5 or 2% (6 or 24 µM dissolved O_2_ at 30 °C, respectively). To study the N_2_O consumption by the bacterium, vials were also supplemented with N_2_O 5% (1.2 mM). A concentration of 10 mM KNO_3_^−^ was also added to the cultures as alternative respiratory substrate as indicated in the text. When needed, antibiotics were used at the following concentrations (in µg/mL): kanamycin, 30; spectinomycin, 25; streptomycin, 25; tetracycline, 10.

### 4.2. Gas Measurements

After transferring aerobically grown bacteria into anaerobic vials, they were placed together with blanks and gas standards in a thermostatic water incubator at 30 °C. Cells dispersion and equal distribution of gases throughout the vial liquid and headspace was achieved by continuous stirring at 700 rpm. Emission of gases (O_2_, NO, N_2_O, and N_2_) resulting from bacterial aerobic and anaerobic metabolism were monitored by automatic gas sampling. Gas measurements were analyzed as described by Bueno at al., 2015, and Molstad et al., 2007 [39,48]. Briefly, the gas samples were drawn from each bacterial culture, and with each sampling an equal volume of He was pumped back into the vials to maintain gas pressure at 1 atm. Sampling and gases’ measurements were performed as previously described in detail [39].

### 4.3. Determination of Bacterial Growth and NO_3_^−^ and NO_2_^−^ Concentrations

To measure bacterial growth and NO_3_^−^ and NO_2_^−^ concentrations, aliquots from the liquid phase of vials were withdrawn manually by using sterile syringes. Bacterial growth was determined by measuring cell density at 600 nm (OD_600_). Concentrations of NO_3_^−^ and NO_2_^−^ were determined as described by Bueno at al., 2015 [39].

### 4.4. Kinetic Analysis from Aerobic and Anaerobic Respiration

Aerobic and anaerobic respiration kinetics were determined as described by Bueno et al., 2015 [39]. To determine O_2_ and NO concentrations in the liquid, we considered the pressure of the gases, their solubilities, and their transport coefficients among headspace and liquid. O_2_ dissolved in liquid was also calculated considering O_2_ respiration rate during bacterial growth (see Molstad et al., 2007 for details). We analyzed N_2_O concentrations as µmol N_2_O vial^−1^, while N_2_ was estimated as net production of N_2_. Growth rates (µ_ox_) and reduction of NOx during the anoxic phase (µ_anox_) were determined by regression [ln (V_e−_) against time] for the phases with exponentially increasing rates. Determination of cells yield (cells pmol^−1^ e^−^) was estimated considering the number of biomass produced per pmol electron consumed by the transport electron chain to reduce O_2_ to H_2_O in the oxic phase (Yield_ox_) or by the denitrifying machinery during the anoxic phase (Yield_anox_). V_max_ tells us about the specific efficiency for O_2_ and NO_x_ respiration per cell. For further details regarding these calculations, see Molstad et al. (2007) [48] and Nadeem et al. (2013) [27].

### 4.5. Determination β-Galactosidase Activity

β-galactosidase activity to investigate gene expression was analyzed as previously described [49]. In brief, 5 mL of cells incubated for 20 and 30 h under the conditions detailed in the text were collected, centrifuged, and resuspended in 500 µL of growth medium. In total, 25 µL of this culture was mixed with 20 µL of freshly prepared SDS 0.1%, 25 µL chloroform, and 100 µL of Z-buffer (60 mM Na_2_HPO_4_, 40 mM NaH_2_PO_4_, 10 mM KCl, 1 mM MgSO_4_, and 50 mM β-mercaptoethanol). Next, 20 µL of ONPG (4 mg/mL) was added to initiate the reaction. Reaction mix was incubated at room temperature before the reaction was terminated by addition of 75 µL of 1 M Na_2_CO_3_. Supernatant was collected and absorbance at OD_420_ and OD_550_ used to determine β-galactosidase specific activity in Miller units.

## Figures and Tables

**Table 1:** Summary of growth parameters from O_2_ (oxic growth phase) and NO_3_^−^ respiration (anoxic growth phase) in the *B. diazoefficiens* 110*spc*4 parental and *fixK*_2_ and *nnrR* mutant strains (**A**) and other parameters observed through anoxic NO_3_^−^ respiration (**B**).

A					
Genotype	Oxic Growth Phase			Anoxic Growth Phase	
	µ_ox_ (h^−^^1^)	Yield_ox_ (cell pmol^− 1^ e^−^)		µ_anox_ (h^−^^1^)	Yield_anox_ (cell pmol^−1^ e^−^)
**110*spc*4**	0.10 (±0.03) a	13.3 (±1.1) a		0.049 (±0.004)	5.1 (±0.8)
**∆*fixK*_2_**	0.055 (±0.008) b	6.6 (±0.3) b		0.00	-
**∆*nnrR***	0.090 (±0.004) a	13.1 (±0.6) a		0.00	-
**B**					
	**[O_2_] at Onset of NO_3_^−^** **Reduction (µM O_2_)**	**Max [NO] in Liquid** **(nM)**	**Fraction of NO_3_^−^ Reduced to N_2_ (%)**	**Final OD_600_** **(oxic)**	**Final OD_600_ (anoxic)**
**Genotype**					
**110*spc*4**	5 (±0.3) a	600 (±400) a	100	0.080 (±0.005) a	0.40 (±0.05) a
**∆*fixK*_2_**	-	-	-	0.044 (±0.003) b	0.042 (±0.002) b
**∆*nnrR***	3.3 (±2.3) b	10797 (±1700) b	-	0.079 (± 0.001) a	0.061 (±0.004) b

All the experimental vials contained an initial O_2_ concentration of 2% at headspace and 10 mM NO_3_^−^ in the growth medium. Data are means with standard error (in parenthesis) from at least three independent cultures. Values in a column followed by the same lower-case letter are not significantly different according to One-Way ANOVA and the Tukey HSD test at *p* ≤ 0.05. Apparent oxic growth (µ_ox_, h^−1^) and anoxic growth (µ_anox_, h^−1^) rates based on O_2_ consumption during the oxic phase or reduction of NO_3_^−^, NO_2_^−^, or N_2_O during the anoxic phase. Yield (cells per mole electron) based on increase in OD vs. cumulated consumption of O_2_ or reduction of NO_3_^−^, NO_2_^−^, or N_2_O. -, not detected.

**Table 2:** Summary of growth parameters from N_2_O consumption in the *B. diazoefficiens* 110*spc*4 parental and *fixK*_2_ and *nnrR* mutant strains (**A**) and other parameters observed through the incubations, depending on the presence or absence of NO_3_^−^ (**B**).

A						
	Anoxic N_2_O Respiration −NO_3_^−^			Anoxic N_2_O Respiration +NO_3_^−^		
Genotype	µ_N20_ (h^−1^)	Yield (cell pmol^−1^ e^−^)	N_2_OR (µmol N_2_ h^−1^)	µ_N2O_ (h^−1^)	Yield (cell pmol^−1^ e^−^)	N_2_OR (µmol N_2_ h^−1^)
**110*spc*4**	0.028 (±0.002)	29 (±8)	4.6 (±0.5)	0.046 (±0.003)	48 (±13)	8.4 (±0.8)
**∆*fixK*_2_**	-	-	-	-	-	-
**∆*nnrR***	-	-	-	-	-	-
**B**						
**−NO_3_^−^**						
**Genotype**	**[O_2_] at Onset of N_2_O Reduction** **(µM O_2_)**		**Max [NO] in Liquid** **(nM)**		**% N_2_O Reduced to N_2_**	**Final OD_600_**
**110*spc*4**	0.66 (±0.05)		-		100 a	0.13 (±0.03) a
**∆*fixK*_2_**	-		-		-	0.06 (±0.01) b
**∆*nnrR***	-		32.2 (±8.8)		8 (±0.5) b	0.05 (±0.02) b
**+NO_3_^−^**						
**Genotype**	**[O_2_] at Onset of N_2_O Reduction** **(µM O_2_)**		**Max [NO] in Liquid** **(nM)**		**% N_2_O Reduced to N_2_**	**Final OD_600_**
**110*spc*4**	0.15 (±0.05) a		113 (±30) a		100 a	0.2 (±0.05) a
**∆*fixK*_2_**	-		35 (±6.9) b		-	0.05 (±0.01) b
**∆*nnrR***	0.8 (±0.3) b		2250 (±85) c		12.5 (±2.1) b	0.07 (±0.01) b

All the experimental vials contained an initial O_2_ concentration of 0.5% at headspace, 5% N_2_O, and 10 mM NO_3_^−^ in the growth medium when indicated. Values in a column followed by the same lower-case letter are not significantly different according to One-Way ANOVA and the Tukey HSD test at *p* ≤ 0.05. Data are means with standard error (in parenthesis) from at least three independent cultures. Apparent anoxic growth (µ_N2O_, h^−1^) rates based on N_2_O reduction. Yield (cells per mole electron) based on increase in OD vs. cumulated consumption of N_2_O without or with NO_3_^−^. -, not detected.

## Supplementary Materials

The following are available online at https://www.mdpi.com/article/10.3390/ijms23031486/s1.
