# Supplementary material for: Regulation of the Emissions of the Greenhouse Gas Nitrous Oxide by the Soybean Endosymbiont Bradyrhizobium diazoefficiens
Source: Int J Mol Sci. 2022 Jan 27;23(3):1486. doi: 10.3390/ijms23031486 (PMC8836242; doi:10.3390/ijms23031486)
Supplement: Supplementary file 1 [file ijms-23-01486-s001.zip › ijms-1545084-supplementary.pdf]

International Journal of
Molecular Sciences

Supplementary Material

A

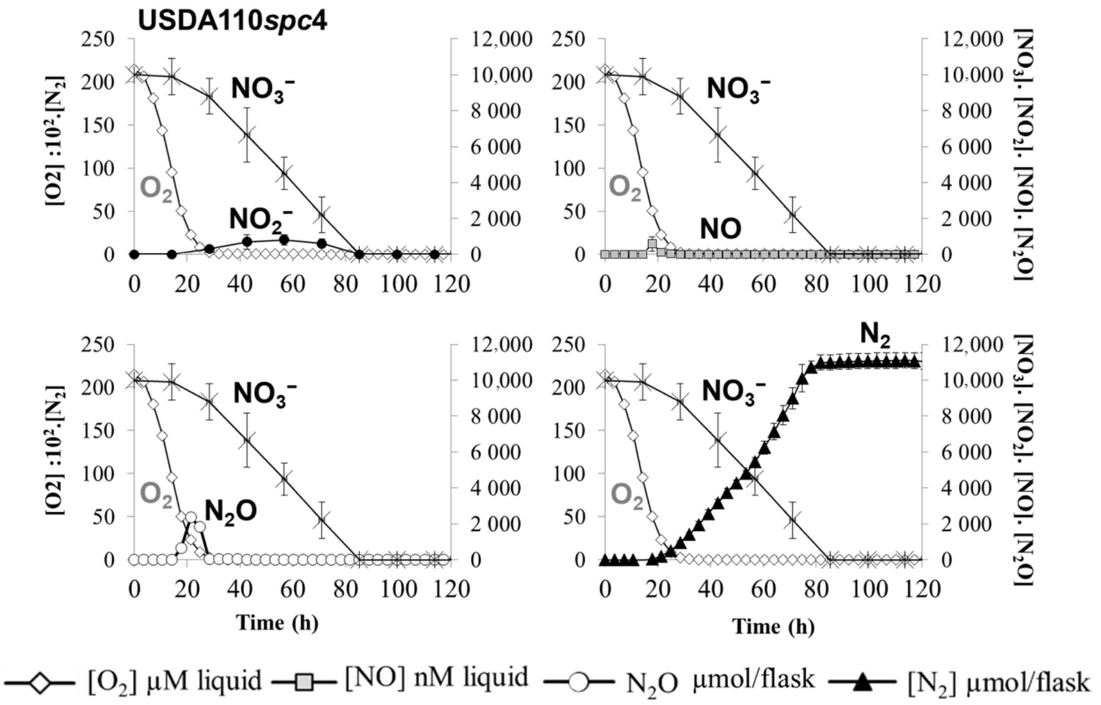

B

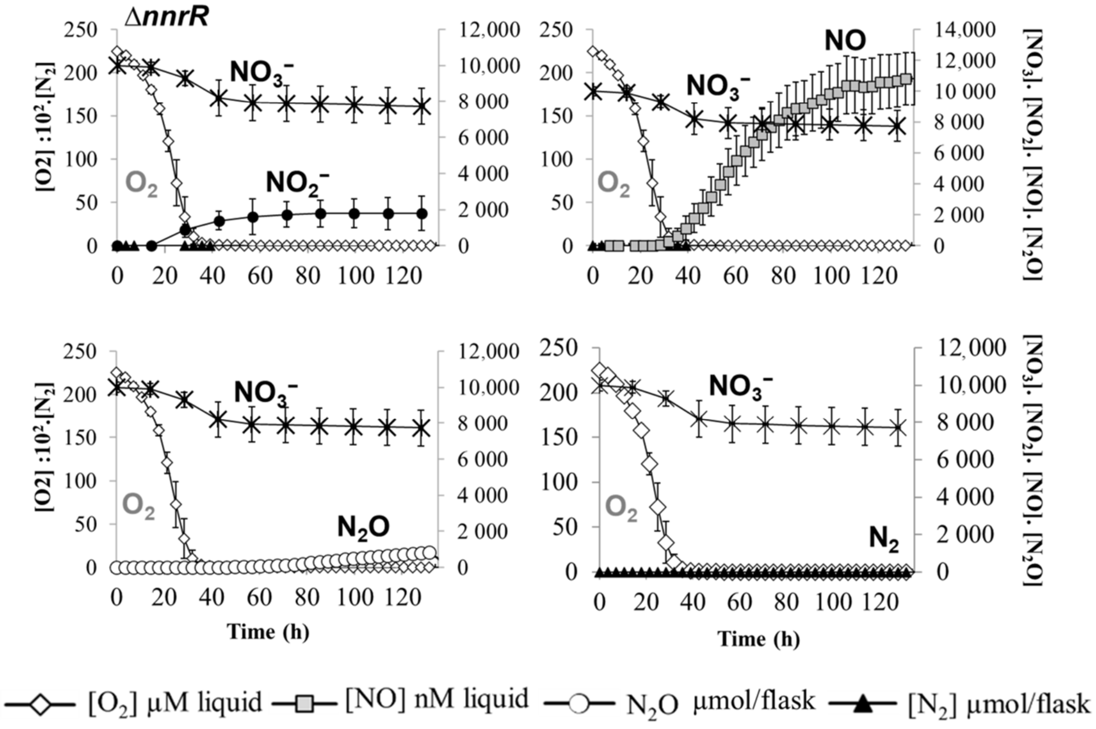

Figure S1. Denitrification phenotypes of the parental strain *B. diazoefficiens* 110*spc*4 (**A**) and the mutant strain Δ*nnrR* (**B**). Measurement of O_2 and NO_3^- respiration and concentrations of denitrifying intermediaries (NO_2^-, NO, N_2O, N_2. Cells were incubated with O_2 2% and 10 mM NO_3^- as oxic and anoxic respiratory substrates, respectively. O_2, and NOx concentrations

were monitored by automatic sampling from headspace and liquid phase. Data are the means and standard deviations of at least three different cultures.

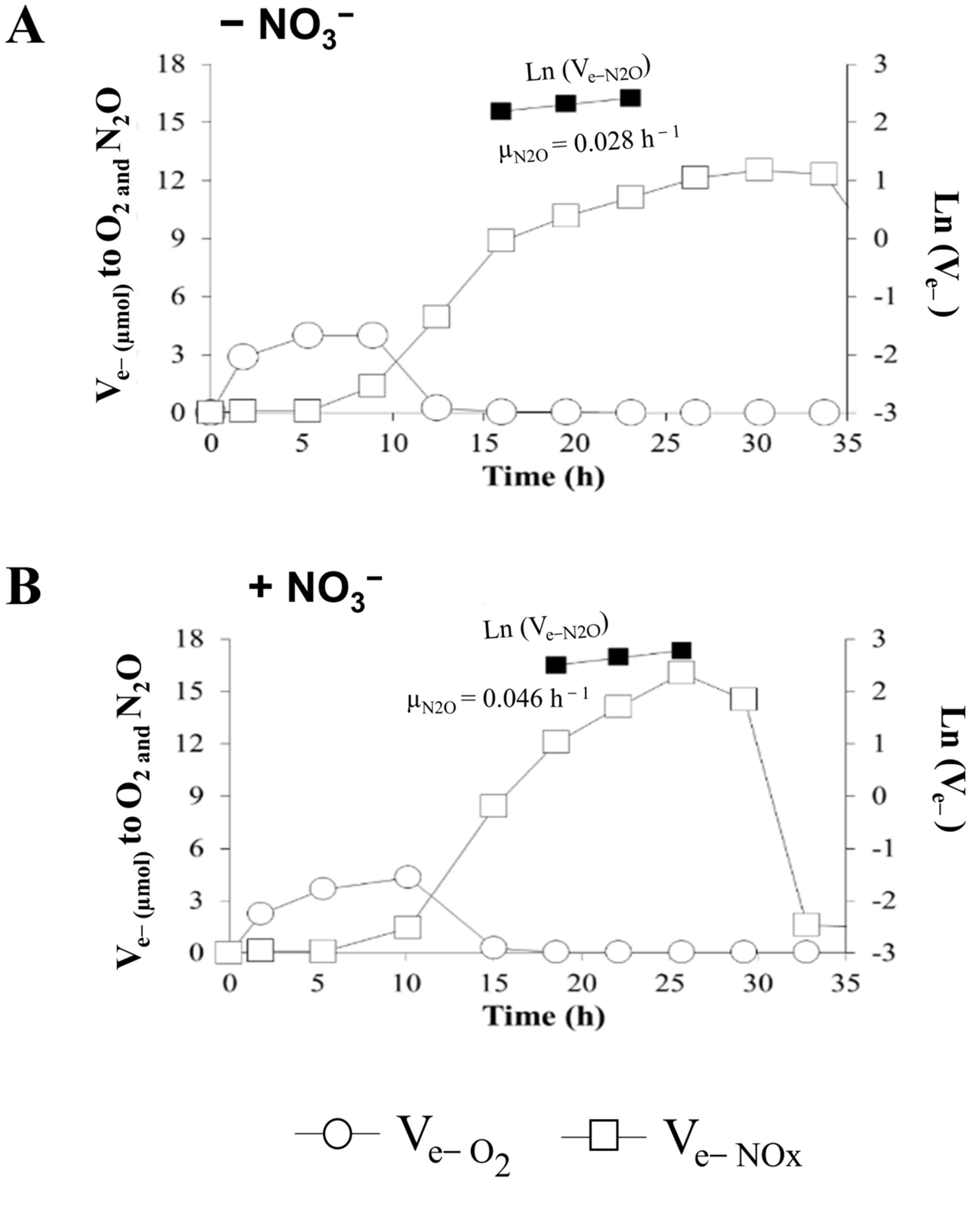

Figure S2. Electron flow analysis N_2O from *B. diazoefficiens* 110*spc*4 in the absence (**A**) and in the presence (**B**) of NO_3^-. Electron flow (Ve^-) to N_2O is shown as log-transformed values for the anoxic phase (filled black square symbols) with exponential increase. Shown is one representative experiment from at least three independent biological replicates.

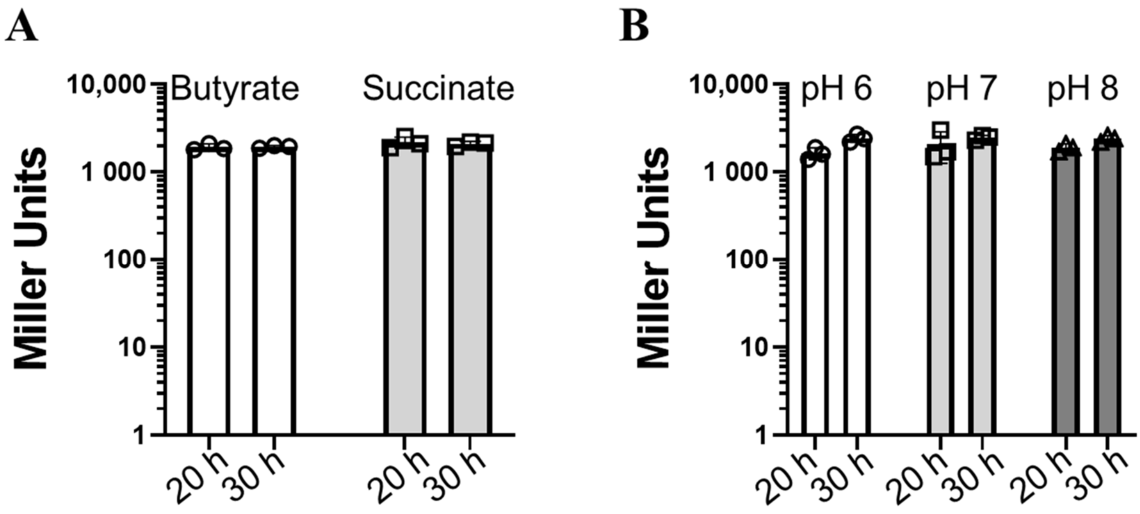

Figure S3. Expression of *nos* genes under different C-sources and pHs. β-galactosidase activity from a *nosR-lacZ* transcriptional fusion chromosomally integrated in *B. diazoefficiens* 110*spc*4. Cells were raised aerobically and then incubated anaerobically during 20 and 30 h with 5% N_2O as anaerobic respiratory substrate in a growth medium containing butyrate or succinate as the sole C-source or under pH 6, 7 and 8. Data are expressed in miller units as average values ± standard error (*n* = 3) from at least 3 different cultures.
